# Poverty proofing healthcare: A qualitative study of barriers to accessing healthcare for low-income families with children in northern England

**DOI:** 10.1371/journal.pone.0292983

**Published:** 2024-04-26

**Authors:** Elaine Bidmead, Louise Hayes, Laura Mazzoli-Smith, Josephine Wildman, Judith Rankin, Emma Leggott, Liz Todd, Luke Bramhall

**Affiliations:** 1 Institute of Health, University of Cumbria, Carlisle, Cumbria, United Kingdom; 2 National Institute for Health and Care Research (NIHR) Applied Research Collaboration (ARC), North East and North Cumbria, Newcastle Upon Tyne, Tyne and Wear, United Kingdom; 3 National Institute for Health and Care Research (NIHR), Population Health Sciences Institute, RSS Specialist Centre for Public Health, Newcastle University, Newcastle upon Tyne, Tyne and Wear, United Kingdom; 4 School of Education, Durham University, Durham, County Durham, United Kingdom; 5 Population Health Sciences Institute, Newcastle University, Newcastle upon Tyne, Tyne and Wear, United Kingdom; 6 Children North East, Newcastle upon Tyne, Tyne and Wear, United Kingdom; 7 School of Education, Communication and Language Sciences, Newcastle University, Newcastle upon Tyne, United Kingdom; University of Leeds, UNITED KINGDOM

## Abstract

Poverty impacts negatively on children’s health and future life chances. Access to the UK’s National Health Service (NHS) is based on clinical need rather than the ability to pay but horizontal inequities in access exist. Children North East, a charity supporting children experiencing poverty, are working with partners to reduce the impacts of poverty on NHS access. This collaborative study aimed to understand barriers to healthcare access faced by families living on low incomes to validate and support further development of a Poverty Proofing© healthcare tool. Twenty-four parents and eight Voluntary Community Social Enterprise sector staff participated in qualitative interviews or focus groups. Data were analysed thematically, and three main themes were identified as impacting access to healthcare: hidden costs, securing appointments and developing relationships with healthcare providers. We conclude that low-income families experience both financial and other barriers to accessing NHS healthcare and that these barriers are exacerbated for low-income families living in remote/rural areas.

## 1. Introduction

Poverty is a major determinant of health and life opportunities that impacts negatively on children’s futures. Poverty occurs when people’s resources are well below their minimum needs [1]. A recent UK report shows that ‘children born into the poorest fifth of families in the UK are almost 13 times more likely to experience poor health and educational outcomes by the age of 17 years’ [2]. Children experiencing poverty are at greater risk of becoming overweight, developing asthma and having tooth decay, as well as performing poorly at school [3]. Poverty also increases the risk of poor mental health [4] and early adulthood mortality [5,6]. Children and young people in the UK report that poverty has a negative effect on their wellbeing and causes feelings of exclusion, shame and unfairness [7].

Access to the UK’s National Health Service (NHS) is based on clinical need rather than an individual’s ability to pay. It is free at the point of delivery irrespective of financial circumstances. However, ’horizontal inequities’ exist in healthcare utilisation [8]. While poorer health in poorer people results in them consuming more healthcare (at every age), richer people tend to access healthcare at an earlier stage and consume more preventive and specialist care [8,9]. In this study, we sought to explore how financial circumstances impact on healthcare access among families with children living on low incomes (commonly defined in the UK as below 60% of the median income [10]).

In 2021–2022, 29% of UK children lived in poverty [11]. In June 2023 the figure was substantially higher (35%) in the North East of England [12]. Poverty is a significant cause of the UK North-South divide in children’s life chances as it limits access to the circumstances that help to ensure good health [13].

Children North East (CNE) is a charitable organisation founded to support children living in poverty. In 2011, children working with CNE identified an end to discrimination in school as their main priority. In response, CNE, with support from the North East Child Poverty Commission, developed ’Poverty Proofing© the school day’. This is an audit and action-plan development tool for schools which aims to remove poverty-related barriers to learning [14]. Following several successful implementations, the North East and North Cumbria Child Health and Wellbeing Network expressed interest in developing Poverty Proofing approaches for healthcare settings. In 2019, the network commissioned CNE to undertake a priority setting consultation exercise with young people and families so that their views could be used to plan and steer poverty proofing within healthcare [15]. The present study, conducted by the universities of Newcastle, Cumbria and Durham in partnership with CNE, aimed to build on that consultation; our intention was to add rigour and depth of understanding to those earlier findings to support further development of CNE’s Poverty Proofing© healthcare action-planning toolkit.

The work was conducted during a period when the NHS was experiencing service pressures due to increased demand from COVID-19, workforce issues (including high sickness rates and understaffing), and the impacts of austerity. Comments during data collection made clear that these pressures had created a situation in which access to health services (such as appointments with dentists, General Practitioners (GPs) and hospitals) was challenging for many people, irrespective of financial circumstance. In this paper we focus on issues impacting access to healthcare that are amplified by low-income.

## 2. Material and methods

The COnsolidated criteria for Reporting Qualitative research (COREQ) [16] were used to guide our research design; our COREQ checklist is attached as S1 Appendix.

### 2.1 Sample and recruitment

Data collection was undertaken with parents on low-incomes and with professionals working in the Voluntary, Community and Social Enterprise (VCSE) sector who support people on low-incomes. We had planned to involve healthcare professionals (HCPs) but this was not viable due to COVID-19.

Parent participants were purposively recruited via VCSE organisations in the North East and North Cumbria. VCSE organisations were supplied with study information sheets which they shared with service users they identified as experiencing low-income (number unknown). Participants were offered a £20 gift voucher to thank them for their contribution. Only six parents volunteered to be interviewed. To increase participation in the study we conducted three focus groups at social/support group sessions run by VCSE organisations in North East England, adding the views of another 17 parents. No parents were known previously to the researchers.

Three VCSE organisations working in health and wellbeing were contacted by email, supplied with a study information sheet, and invited to contribute to the research. Organisations were offered compensation of £20 per person participating. Eight staff members volunteered to participate. All were based in North Cumbria and two were known to EB previously.

No participants dropped out. The number of parents/VCSE staff choosing not to participate is unknown.

### 2.2 Ethics

Ethical approval for the study was granted by Newcastle University Research Ethics Committee (Reference: 2236/15258 Date: 23/11/2021).

All participants were provided with written and verbal information about the study and invited to ask questions prior to participating; all provided written informed consent before taking part.

### 2.3 Research design and setting

The research design is qualitative with a focus on barriers to healthcare access encountered by low-income families to support understanding of how participants experienced these barriers. Qualitative research is particularly useful when seeking to understand participants’ perspectives [17]. We used interviews and focus groups to situate this understanding in the lived experiences of our participants and to elicit responses from within participants’ own contexts—rather than assuming that knowing what a barrier is equates with understanding how it is experienced.

Topic guides were devised to structure and facilitate in-depth one-to-one interviews and focus groups. The parent topic guide was piloted with two parents and no amendments were made (attached as S2 Appendix), the VCSE guide was not piloted (attached as S3 Appendix). Both topic guides prompted participants to think about different aspects of healthcare, including getting appointments, accessing appointments, emergency situations, and staff attitudes; it also allowed participants to introduce issues significant to them in addition to those covered by the guides.

The research was undertaken by EB and LH. Both are female, mid-career researchers (educated to PhD level) trained and experienced in qualitative data collection methods and analysis. Researchers shared with participants their names, job roles, qualifications, and reasons for doing the research.

One round of data collection was undertaken. Parents volunteering for interviews were given the option of in-person, telephone or video; five opted for video which were conducted using Microsoft Teams or Zoom by LH and one opted for telephone, conducted by EB. Interviews lasted between 15 and 60 minutes and were recorded and fully transcribed. Three parent focus groups were conducted with established groups in community venues and facilitated in person by LH (n = 6, n = 2, n = 9). Parent focus groups lasted between 40 and 60 minutes; two were audio-recorded and fully transcribed; notes were taken in the third at the request of participants. The interview and two focus groups with VCSE staff were conducted by EB via online video using Microsoft Teams, they lasted between 60 and 80 minutes and were recorded and fully transcribed. No others were present during data collection besides the researchers and participants. Transcripts were not shared with participants.

### 2.4 Timescale

Data were collected between December 2021 and March 2022.

### 2.5 Analysis

Anonymised transcripts were subjected to ‘practical thematic analysis’ [18]. Following familiarisation with the data, a sample of six transcripts were independently line by line coded by two researchers (EB and LH), inductive codes emerged from the data; deductive codes aligned with the topic guide. The two researchers then met to discuss codes and initial themes. All transcripts were then coded by one researcher (EB) using NVIVO; a coding tree is available as S4 Appendix. Following coding, coded data and initial themes were critically discussed at team meetings and the main themes were agreed. EB and LH drafted the paper which was reviewed by JR then commented on and agreed by the study team and finalised.

## 3. Results

Semi-structured interviews were conducted with six parents (all mothers) aged approximately 20–60 years, four of whom were lone parents. Seventeen parents (16 mothers and one father) aged approximately 20–40 years, participated in one of three focus group discussions. As participants were purposively recruited because of their financial circumstances we did not collect further demographic or socioeconomic information from them. Seven staff took part in focus group discussions and one in a semi-structured interview; all were female and aged over 30 years. The results presented below include quotations to provide rich and faithful accounts; each transcript was given an identity code to indicate whether data came from a parent interview (ParentInt), parent focus group (ParentsFG), VCSE interview (VCSEInt) or VCSE focus group (VCSEFG

Three deductive domains and three inductive themes were identified during analysis (detailed in Table 1). VCSE participants were asked about the challenges of living in poverty and several parents commented on this topic, we summarise these responses first. Next, we present inductive thematic findings. We identified three main themes: hidden costs, securing appointments and developing relationships with healthcare providers. We then present domain summaries on participants’ awareness of sources of financial assistance for accessing healthcare and their suggestions for improvements.

**Table 1:** Thematic barriers to healthcare access.

Barriers to access	Theme type	Summary
Challenges from living on low-incomes	Deductive domain	Causes stress, embarrassment and stigma, and has layering and cumulative impacts on people’s lives (health; choices; access to services and support).
Hidden Costs	Inductive theme	Including costs of transport; subsistence in hospitals; parking; loss of income from employment
Securing appointments	Inductive theme	Caused by poor digital access; lack of GP/dental appointments; appointment booking systems; appointment times; difficulties in navigating health systems; unavailability of childcare
Relationships with healthcare providers	Inductive theme	Caused by lack of continuity in care; health service pressures; social distance between healthcare professionals and patients; poor communications and information; healthcare professionals lacking awareness of the reality of life on a low-income
Awareness of financial assistance	Deductive domain	Poor awareness of assistance due to poor provision of information and information lacking detail on eligibility
Suggested improvements	Deductive domain	Better provision and quality of information on financial assistance; assistance with subsistence costs in hospitals; system changes including pre-bookable appointments and flexibility around times; better training for HCPs to raise awareness and understanding of impacts of poverty on families

### 3.1 Living on a low income

Some parents shared the challenges of living with financial hardship. It was typically described as hard, stressful, embarrassing and stigmatising:

“It’s a big struggle on a on a day-to-day basis… It’s like this week it’s my, my poor week as I call it, I get like £63 today to last me a week and out of that I’ve got my bills to pay, my food to pay … it’s just impossible to live on.” (ParentInt1)

Participants in ParentsFG1 spoke about the embarrassment felt at having to ask for assistance due to financial hardship. ParentInt2 shared the stigmatising effects of poverty: “stigma about people claiming benefits, and ‘*Oh*, *you’re useless*,’ and, ‘*You’re timewasters and scroungers*.*’*” They stressed that they had always worked until their child was born with significant health needs and explained that they were “quite proud”; being asked to prove they were in need was highly embarrassing to them, for example:

“You either have to print off a statement, which to me is embarrassing, because it’s got every bit of how they break down how much you’re going to get paid … the other thing that I find quite embarrassing is, if, for example, my son’s admitted into hospital, … they’ll ask me about what our situation is. Do I work or don’t I work? And do we have social worker? … And I feel that’s quite probing … I think there’s a lot of things that could be improved on, to make you feel not as scummy about the way you are.” (ParentInt2)

VCSE participants described what living in poverty meant to them. Descriptions highlighted both absolute poverty (reliance on food and clothing banks) and relative poverty (having to do without luxuries, holidays, family days out etc.). Living in financial hardship was thought to impact people’s long-term health and resilience due to poor diet and limited social engagement (VCSEFG1). VCSE participants also emphasised the stress caused by poverty and the complex, layering and cumulative impacts it has on families, which can become overwhelming.

“Poverty … It’s an overwhelm with lots of different factors that eat you away, until you feel like you have few options.” (VCSEInt1)

“It’s bad enough if you have a child with a physical need … If you add on the fact that if there’s poverty and you’ve got a battle with the health system … you know it’s all about those layers.” (VCSEFG2)

Like parents, VCSE participants highlighted people’s embarrassment about their financial situations because “families often feel judged” (VCSEFG2). Consequently, some attempt to hide or disguise the effects poverty has on their lives.

VCSEFG2 pointed to situations where the impacts of poverty may be misconstrued and understood instead as neglect. For example, one participant highlighted the practice of setting targets for parents during Child Protection or Child in Need Plan meetings, such as attending health appointments. They noted “if you weren’t actually able to access the appointment because of difficulties getting there” then that may be seen as the parent not complying:

“Some families will go down the I can’t afford it. Others won’t say that because they’ll be fearful of ‘*Well*, *if you can’t afford to go to health … what’s happening there*’ and they will come over as disengaged and difficult when actually they can’t, they cannot, actually manage to get there because of finance.” (VCSEFG2)

### 3.2 Hidden costs–financial barriers to accessing healthcare

#### 3.2.1 Transport

Transport costs emerged as a major barrier to accessing healthcare for low-income families. Most could access GPs locally and so could attend relatively easily, but attending hospital appointments often involved longer journeys and several buses if undertaken by public transport, or excessive parking fees if undertaken by car.

Participants indicated numerous times that the costs of transport to appointments were a concern to them and these costs increased exponentially for parents with multiple appointments, living in remote/rural areas, or having to travel long distances to access secondary care. There were also significant time implications for parents living in remote areas without access to a vehicle, one parent from a rural village elaborated:

“It’s about 25, 26 mile away so like I say to get to [large town], you’ve either got to get a taxi … to [small town] and then a train … to [large town] … or get a bus which takes about 45 minutes. The train takes about 10 minutes, but they don’t run as often … For a taxi into [small town] its £15 each way … The train I think it’s about £7 or £8 return now … for both of us, you’re looking, by the time you get taxis and that, you’re looking at 50 odd pound before you even get to the appointment […] So, you have to cancel appointments or work around it.” (ParentInt1)

In remote or rural areas, people may be required to access secondary care at sites across the region, irrespective of where they live. Where people are referred is determined by the availability of appointments. However, people on low incomes face the dilemma of covering significant costs or delaying their healthcare:

“Where health authorities are trying to squeeze you in for an appointment and not make you wait, they will offer you say [hospital name] for example … for some families, that’s easy enough [but] often it’s a choice of getting yourself over to another hospital … taking the day off work or waiting maybe a month or two, you know, for your child to be seen, which is a really difficult decision to make […] they say you aren’t forced to go there, you can wait… so that while you’re not denied healthcare it will be delayed, not through your lack of engagement, not through you not putting your health or your children’s needs first, it’s because of the affordability of the offer of what your appointment looks like.” (VCSEFG2)

When it comes to accessing tertiary care, such challenges are exacerbated. VCSEFG1 commented upon the number of people in North Cumbria having to travel “out of county” for treatment. For example, for specialist children’s services, “Newcastle’s the closest or … it’s Manchester” (VCSEInt1). This entails expensive and time-consuming travel and potentially lost earnings if time off work is necessary. One parent in rural North Cumbria highlighted the journeys involved in accessing care for their child ‘out of county’:

“I went to see [consultant], so of course that incurred money for going across to [hospital in Newcastle], and then he asked for an MRI scan, which [hospital in North Cumbria] did, and then he sent me an appointment to have an MRI scan done with contrast, but at [another hospital in Newcastle].” (ParentInt2)

This means some people simply cannot afford to attend hospital appointments, a situation that is compounded where multiple appointments are involved.

#### 3.2.2 Subsistence during hospital attendance

Paying for food and drinks during hospital attendance emerged as a significant challenge for parents who could not afford the costs involved; this was an issue when children were outpatients and inpatients. Parents highlighted the limited options within hospitals to buy food, and stressed the expense of hospital food outlets in the North East and North Cumbria:

“Hospital food is not affordable … I just had to find the money. My mam would help us out a lot. My daughter’s grandad would help. But it is still too much to pay back.” (ParentInt5)

“Even getting a cuppa, it’s like a fiver in the hospital.” (ParentsFG2)

Costs increased when children were inpatients. It was reported that no food is given to parents staying with a child, no matter their age–unless a mother is breast feeding. One parent had experienced extended hospital stays when their baby was born with health complications. They stayed in charitable accommodation at the hospital; this provided cooking and laundry facilities. However, they did not regularly use these due to financial and time pressures which then impacted their ability to eat healthily; they reported missing meals and not looking after themselves properly. ParentInt6 noted: “The food in the hospital, it’s not conducive to low cost” and reported “living on Subway sandwiches and Pot Noodles but I can’t see it being nutritionally balanced” and that “I try to pack snacks for both of us”.

Whilst some preparedness may be expected for planned admissions, this is not possible with emergency admissions which were reported to trigger even more expenditure. VCSEInt1 highlighted the realities of emergency admissions for parents:

“As we know from our stats, that’s likely to be an ambulance admission. So, they’re less likely to have what they need with them. They’re less likely to have the people that they need with them […] you’d be lucky if you find a vending machine. You’d be lucky if you’ve got the right change on you for the vending machine and you’d be lucky if the vending machine’s got anything in it other than tea or coffee. I think you might find that a kind nurse might offer you a cup of tea and a piece of toast, if they’re not rushed off their feet.”

This scenario was confirmed by parents, for example:

“I didn’t have a budget for when he got rushed into hospital, because nobody knew it was going to happen. So, then I was borrowing money and things like that, just to travel over there and back.” (ParentsFG1)

On a positive note, two parents mentioned specific nurses or wards being helpful in terms of providing food and drinks, but this was dependent on the actions of individual staff members rather than hospital policy.

#### 3.2.3 Discharge from hospital

Participants highlighted the costs associated with discharge from hospital following an admission by ambulance. This is a particular issue when the emergency department is a significant distance from home and if discharge occurs when public transport is not operating. Hospital discharge “could be any time of the day; it could be at any time of the night” (VCSEFG2) and “Your only option for getting home without any transport would be a taxi” (VCSEInt1). Parent5 described attending emergency departments several times; each visit requiring a taxi. Sometimes their child would be admitted, but at others they would give medication and discharge them, “so that would be another £30 taxi fare back home” (ParentInt5).

#### 3.2.4 Parking costs

For parents who owned a vehicle parking charges were a concern; they were said to be around “£2 or £3 an hour” which “obviously you don’t have” (ParentFG3). This view was repeated by VCSEFG1 where it was mentioned that “parking is always an issue because of the extra costs parking brings” which caused stress “because you’d never know how long” you will need. ParentInt2, whose child was in hospital for several weeks, reported high parking charges when their partner drove from North Cumbria to Newcastle at weekends. After a time, somebody mentioned cheaper parking that could be used but this involved using a shuttle bus to the hospital, which took up time and was not available at weekends.

#### 3.2.5 Impacts on income

Some participants spoke about lost income due to attending appointments with children. ParentInt2 reported losing pay from having to miss work when their child was ill because they did not “qualify for sick pay”. A contributor in VCSEFG2 highlighted that some families cannot take time off work because it will affect social security payments.

For others, having an ill child had resulted in them losing employment. Participants in VCSEFG2 talked about several parents who gave up employment because they found working whilst caring for an ill child impossible and decided it was “too much to be able to juggle all these things and meet my child’s needs”. Another added that “the way that health services shape themselves impacts” because people must choose between “working or putting children first”; they highlighted “the amount of negotiation you have to do to try and get not every single health appointment in the middle of the day. You know, it’s, it’s impossible” (VCSEFG2).

### 3.3 Securing appointments

#### 3.3.1 Digital first

The issue of digital access to health services was raised on several occasions, as was the expectation that everyone has a smart phone or internet access with unlimited calls/data. Parents questioned if people on low incomes did. For example, whilst some participants at ParentsFG3 noted the easiest way to get a GP appointment was via an app, one shared that they did not have a smart phone nor access to a computer.

VCSE Participants referred to a digital divide wherein “you are less likely to have a laptop if you don’t have the money” (VCSEFG1). COVID-19 was seen to have accelerated the move to digital first services, which many people have adapted to over time, but “other people haven’t been able to just because they can’t afford it” (VCSEFG1). A participant in VCSEFG2 pointed to the requirement to have a mobile phone to receive a code that enabled one to access lateral flow tests for COVID-19. VCSEInt1 noted that:

“We absolutely know that the majority of people [experiencing financial hardship] need face to face engagement because they need to build up trust with their caregiver and … we do know that a lot of people will avoid digital connection and will only go for face-to-face connection because that’s the way that they work. That’s the way they’ve always operated.” (VCSEInt1)

#### 3.3.2 GP appointments

Whilst not directly related to family poverty, almost all participants described difficulties in securing GP appointments, for example: “not being able to get appointments when you need them… waiting weeks and weeks, and weeks” (ParentsFG1). Others reiterated this experience; one “waited two months” for a GP appointment regarding "a water infection” by which time “it was a kidney infection” (ParentsFG1). VCSE participants also highlighted the difficulties experienced by service users in getting appointments due to “massive waitlists, wait times” (VCSEInt1).

Having to telephone the surgery for appointments was highlighted as problematic, participants described waiting on hold for long durations or having to call multiple times:

“I was in the queue for half an hour or 45 minutes. And then she said, ‘*It’s really busy*, *I can’t give you an appointment*, *you can try tomorrow’*.” (ParentInt4)

“You’re having to ring 30 to 90 times to get through … I think the record is 124 to get an appointment one morning.” (ParentInt6)

Specific issues with telephone consultations were also reported. Participants resented having to wait in for doctors to telephone as “they won’t give you a time for them ringing you back … you’ve got to be free all day. If you miss that day, then you’ve really had it” (ParentsFG2). ParentInt1 reported similarly and commented “It stops me from ringing the doctors more times than enough to be fair” (ParentInt1).

Two important factors emerged for low-income families alongside these issues. First was the cost of being on hold for extended durations. VCSE participants pointed to an assumption that all patients have access to free calls on their home or mobile phones and have data for online access, which is not the case for families facing financial hardship, who are more likely to be on a basic landline tariffs or pay as you go and have limited data on their mobile phone.

“If it’s ringing, it’s free and people just assume that people don’t want that, and they don’t want to be ringing, they want to be in a queue. But … that starts the pennies ticking for people who have to pay for their calls.” (VCSEFG2)

The second issue to emerge was that these difficulties discouraged people from trying to obtain appointments, potentially exacerbating conditions, with some choosing to access Accident and Emergency Departments (A&E) instead:

“They’re not even bothering … because they can’t get through to them. So, they won’t even bother ringing, they won’t ring for the children, they won’t ring for their husbands and won’t ring for themselves anymore and they will go, if it’s an emergency, they would go to the hospital. But apart from that they’ve not even tried, they’ve just stopped ringing because they don’t, they just don’t see the point anymore.” (VCSEFG1)

“The system seems quite complex to navigate, sometimes you’ve got to ring up and then listen to something … some of my families find that really difficult, and they find it so frustrating. I’ve actually got a family who are more likely to ring, do a 999, you know, call a blue light, than actually, you know, ring through for … a GP appointment.” (VCSEFG2)

Participants in VCSEFG1 also highlighted issues in getting appointments for those working more than one job, non-standard hours or with inflexible employment terms who might not be able to take time off work, and for whom time off may result in lost income. Consequently, parents had not attended appointments “because they just couldn’t get time off work” (VCSEFG1).

#### 3.3.3 Dental appointments

The difficulties of accessing NHS dental care are a national problem that has been highlighted extensively in national media in recent months. Indeed, many people have little option but to access dental treatment privately “but that’s not the case for people that don’t have the money to do that” (VCSEFG1). However, even NHS treatment is expensive to those on low incomes. ParentInt5 explained that charges might prevent them from seeking treatment: “if I had to pay for that extra treatment, I wouldn’t get it done” (ParentInt5).

Moreover, participants in ParentsFG3 reported routinely waiting 12–18 months for appointments for their children, during which time their oral health had deteriorated and children were in pain. This scenario was repeated in ParentsFG2, for example: “Trying to get an appointment at the dentist is like a needle in a haystack. You can’t get one”; “they said I couldn’t get an appointment until next year”.

One VCSE contributor in VCSEFG1 suggested that poor dental health is once more becoming a marker of poverty.

#### 3.3.4 Navigating and negotiating appointments

VCSE Participants commented on the difficulties some families face in navigating and negotiating health appointments. The healthcare system was described as a “minefield” involving complicated referral systems and criteria, and what often feels like arbitrary decision making. The system was said to be challenging to negotiate even for those with knowledge of it; for families without such knowledge, it can be debilitating and exhausting, as well as expensive and resource intensive due to having to make multiple telephone calls and searches for information:

“It feels like they’re having to jump through the hoops … they might be feeling are we at the bottom of the pile? Or is it just because of COVID? Or is it just that, you know, our needs are being ignored.” (VCSEFG2)

VCSEInt1 highlighted people’s inability or reluctance to advocate for their healthcare and that of their families as well as poor awareness of what is available to them “so they’re not even accessing those support and services. You know they’re not asking for the referral.” VCSEInt1 ventured that few of us are “good at asking for help when we think we should” and most of us “leave it too late,” but where there are poverty issues “you leave it even later”.

#### 3.3.5 Appointment times

Parents commented on the difficulty of getting children to appointments due to having no control over appointment times. Appointment times determined by healthcare providers often clashed with other responsibilities, such as work or taking other children to school. Some preferred appointments outside the school day to avoid missed education for children, but others preferred appointments during school times so they did not have to take the whole family with them.

ParentInt6, whose child was on a child protection plan, spoke about how hospital appointments could sometimes clash with requirements of the plan, but hospital appointments were not easy to change, “a lot of them are … an afternoon clinic once a month or you have to go to this hospital 16 miles down the road”. VCSEInt1 also commented on the difficulty of altering hospital appointment times, even where long journeys are involved, “I think you’re entirely at the mercy of what comes through on the letter.”

#### 3.3.6 Childcare

Parents talked about challenges in finding suitable childcare so that they or their other children could attend appointments. Participants in ParentsFG1 talked about the possibility of using breakfast or after school clubs, but this was more expense. Moreover, several parents reported not having anyone readily available to provide childcare. This situation was recognised by VCSE Participants who reported that childcare impacted access to healthcare for parents and children alike. Both VCSEFG1 and VCSEFG2 referred to whole families presenting at accident and emergency departments because they had no one to leave children with.

### 3.4 Relationships with healthcare providers

Some parents commented on difficulties they had experienced in forming trusting relationships with HCPs. Not receiving care consistently from the same HCP and having to keep “explaining yourself over and over” was a particular issue for parents in ParentsFG3. Parents offered little comment on healthcare providers’ responses to poverty beyond recognising and valuing moments where healthcare staff went out of their way to be helpful–for example, in the provision of food and drinks.

All VCSE Participants were supportive towards healthcare staff and acknowledged the pressures facing the NHS, for example: most people’s experiences of “health professionals have been positive; most people go into the job for the right reasons and are kind, caring and helpful” (VCSEInt1). Nonetheless, VCSE Participants held concerns over how healthcare providers respond to poverty: “organizationally there are obviously some issues” (VCSEFG2).

Participants in VCSEFG2 contemplated whether clinical staff were trained or supported to think explicitly about poverty; in their experience, where solutions to poverty issues had been presented it had been “individuals who’ve done that as opposed to a systemic approach”. VCSEInt1 pointed to some HCPs that are “authoritative; don’t really listen” which they felt was potentially related to “training issues and recruitment issues in medical school” in that so many “medical students are from private school” and that “GPs tend not to do their placements in deprived areas” (VCSEInt1).

A contributor in VCSEFG2 pointed to an expectation within health services that patients can comply with available provision and when they cannot it is seen as a problem with the patient rather than the system. Another provided an example of when a single parent with four children did not take one of them to an “urgent appointment” because they had no childcare for the others. Whilst the parent had been supported to rearrange the appointment their decision prompted involvement from other services:

“So, [they] felt judged that, [they were not] able to get to that particular appointment at the correct time [because they] had made that decision that, actually, I can’t leave my children alone, and I’m going to have to stay with them.” (VCSEFG2)

VCSE Participants also pointed to issues with communications between patients and healthcare providers; they perceived that access to healthcare information was largely determined by an individual’s ability to navigate and communicate with the system, which was particularly challenging to people with poor literacy, health literacy and digital access/skills. VCSEInt1 highlighted the lack of services to support people who are “overwhelmed or just don’t quite understand or can’t cope”.

### 3.5 Awareness of financial assistance for health-related costs

There was a general lack of awareness about sources of financial assistance for health-related costs. The provision of such information appeared arbitrary, with some people gaining awareness by good fortune and others never becoming aware of it. Participants in ParentsFG3 commented about only finding out about support services and financial help by chance. ParentInt1 had “no idea about anything” and could not recall it ever having been mentioned. However, the idea of applying for assistance appeared burdensome: “If you’ve got to think about support on top of that to get to doctors and stuff like that, it’s another thing you’re having to constantly think about” (ParentInt1).

In contrast, ParentInt6 was aware that hospital transport might be available “but you have to be ready two hours before and you could be waiting up to two hours after”, which is not ideal when taking children. However, they reported that “if you ask about the transport, they can be quite shifty with you, especially if you’re young… we need to keep it for the older generation, as I’ve been told” (ParentInt6).

Participants in ParentFG3 described how they found out that they were eligible to financial support with attending healthcare services “only by chance”.

VCSE informants held little awareness of how to access financial support for health-related costs. VCSEFG1 pointed to “the amount of time it takes to look for the resource and support you can access” and commented that people “just don’t have the time to do that because you’re too busy trying to live your life; trying to work”.

### 3.6 Suggested improvements

Several suggestions for how to improve access to healthcare for families living on low incomes were made.

#### 3.6.1 Financial and practical assistance

ParentInt2 commented on awareness of help with financial costs for healthcare. They stated that had they known they were entitled to help due to low-income then they would have claimed but suggested that although they had read about assistance “in one of the leaflets, for one of the hospitals”, they were not sure whether they were entitled because “it doesn’t say how, it doesn’t say in what circumstances”. Therefore, ParentInt2 felt that information on help with healthcare costs should be included in letters sent out by healthcare providers and Universal Credit, something that says, “If you’re on this benefit, you can be entitled to this.”

Other parents suggested ways to help with subsistence. There were many comments about how hard and humiliating it could be to ask for financial support or for basic necessities to be provided when parents had to stay in hospital with children. Participants in ParentFG1 discussed being “embarrassed to ask” for things that would help but felt this would be easier if things were offered; they thought services should “offer, and overly offer.” ParentInt6 suggested providing vouchers to use at food outlets.

VCSE Participants all wanted increased awareness of financial support for healthcare costs. Participants in VCSEFG1 identified knowledge about financial assistance to be a “big problem”. VCSEInt1 believed that at the very least, people should be made aware of available financial support, but also about any flexibility there may be around appointment times which would make attendance cheaper and more achievable. VCSEFG2 suggested awareness of assistance was so low, even amongst VCSE staff, that research was needed to clarify “what support is available” and for it to become “habit that health providers include that in their letters”. Both VCSEFG1 and VCSEFG2 suggested having leaflets and posters in areas of high footfall, schools, GP surgeries and all healthcare settings. Another contributor, in VCSEFG2, mentioned having seen posters on the doors of supermarket toilets which said “if you struggle to pay for sanitary products just go to customer service and ask for Sandy or something like that”. The group believed something similar would work in healthcare settings.

#### 3.6.2 System changes within healthcare

VCSEInt1 wanted to see more flexibility around appointments times:

“Because at the moment we just feel lucky if you get an appointment … it’s such a battle. But … if you’ve got a long journey, and especially if you’ve got other caring responsibilities or employment responsibilities, the more flexibility the better.” (VCSEInt1)

VCSEFG2 felt that patients should be able to “interact in the way that the doctor’s surgery wants you to at no cost.” They also wanted pre-bookable GP appointments made available to families, which they thought important for planning transport, especially for fitting appointments around the school day and other children’s care. Furthermore, for parents whose children have health conditions, getting GP appointments can feel like a constant trial so pre-bookable appointments would ease their stress: “you know they’re going to be going to the doctors in a month’s time. So why not?”

VCSEFG1 believed improving access and care for low-income families required “a whole cultural system shift”. VCSEInt1 highlighted the social and cultural (class) differences between senior HCPs (i.e. GPs and consultants) and the communities they serve; they wanted medical students to experience more placements in deprived areas. In a similar vein, VCSEFG1 called for improved training for medical staff: “raising awareness about all these different kind of groups that don’t get access and why they don’t get access.” VCSEFG2 felt similarly, “training is really important … specifically about poverty and how that affects families.” VCSEFG2 admitted to having little awareness of how much training in poverty issues different HCPs receive and acknowledged that some professionals are “seeing those issues all the time and [are] really aware of that”. Nevertheless, they felt training and awareness to be very important.

## Discussion

The findings from this study highlighted a range of barriers to accessing healthcare for low-income families. Some were financial, with participants indicating a variety of hidden costs which they found difficult to meet (e.g. transport, childcare, subsistence). Financial hardship was also reported to have other layering and cumulative impacts on families including causing people to feel ashamed, negatively judged, stressed, and stigmatised. Simultaneously, illness caused financial hardship as disposable income was eaten away by these hidden costs. Some reported loss of earnings, others were reported to have lost employment as the demands of caring for a sick child whilst working proved too much. Furthermore, VCSE participants noted the difficulties faced by some families in navigating and negotiating the healthcare system due to its complicated referral systems and decision-making processes, attitudes of HCPs and people’s reluctance or inability to advocate for themselves and their families.

There is a growing literature of the concept of ‘candidacy’, originating with Dixon-Woods et al. [19]. They proposed that access to healthcare is ‘jointly negotiated between individuals and health services’ through a series of complex processes. MacDonald et al. [20] summarise the candidacy process as follows:

‘Individuals must first identify Candidacy by appropriately appraising symptoms, which in turn will inform their decisions around which services may meet their needs. Accessing the services requires navigation of the system. An awareness of the services on offer and the employment of practical resources, such as transport, or the need to contend with disruptions (e.g. time off work), are also required. Individuals’ appearance at health services ‘involves asserting their claim to candidacy’ by providing an accurate description of the problem in order to justify its appropriateness to health professionals. Individuals then experience adjudications from health professionals who decide the suitability of the candidate and the problem. All such negotiations occur in a healthcare culture where some services are more permeable than others.’

However, candidacy is compromised by low-income [19]. Engaging with the healthcare system can be challenging for disadvantaged people due its complexity [21,22] and them personally lacking financial resources (to cover indirect costs); time (due to other pressing commitments—e.g. caring and employment); knowledge/information; and family/social support [22]—lack of family/social support was most apparent here with regards lack of access to childcare to enable attendance at appointments. Furthermore, candidacy can be diminished by health conditions themselves, especially those that reduce agency and self-esteem, such as depression [23]. It is also undermined where multiple disadvantages (such as age, ethnicity, gender, poverty) intersect [24].

Candidacy is further compromised by the permeability of health services, that is ease of accessibility [19]. A&E departments are seen as the most accessible, followed by GP appointments [19,20,25]. In this study participants made clear their difficulties in getting GP appointments and the amount of work required to secure one. Such challenges were reported to have discouraged people from trying to obtain appointments, potentially exacerbating conditions, and resulting in some choosing to access A&E instead.

Concern is expressed over high and/or clinically unnecessary use of A&E, both of which are associated with socioeconomic vulnerability [25,26]. However, McHale et al. [26] found that the odds of ‘inappropriate attendances’ decreased with increasing deprivation. Notwithstanding Dixon-Woods et al. [19] argue that ‘people in more deprived circumstances are likely … to recognise candidacy as a series of crises’, consequently, as crises demand urgency, A&E attendances will be inevitable when access to GP appointments is diminished. Behrens et al [25] report that in their study the inability to secure a GP appointment accounted for 32% of non-urgent attendances at emergency departments. O’Cathain et al. [27] identified six underlying mechanisms of clinically unnecessary A&E attendance (see Table 2), many of which apply here. When these mechanisms combine people are more likely to opt for A&E over other services. They argue that ‘clinically unnecessary use of emergency and urgent care may be judged rational and reasonable once the details of each person’s situation are understood’ [27].

**Table 2:** Mechanisms leading to clinically unnecessary use of emergency and urgent care^a^.

Mechanisms	Description
Risk minimisation	Anxiety over seriousness of symptoms; previous traumatic experiences; consequences of doing nothing–especially when children are unwell
Need for speed	To access immediate pain relief (for self or children); to attend to or resume responsibilities (childcare, education, work); or having waited long enough for symptoms to improve
Convenience	Low treatment seeking burden due to inability to cope–caused by complex, stressful lives, people having multiple responsibilities and too few resources, including time
Compliance	Having been advised by others (family, friends, HCPs)
Consumer satisfaction	A&E giving access to desired tests (e.g. x-rays) and expertise
Frustration	Having attempted but failed to obtain a GP appointment.

^**a**^ **Adapted from O’Cathain et al. 2020 [27].**

COVID-19 weakened candidacy and permeability significantly. Pandemic restrictions accelerated the move to digital healthcare which had the paradoxical effect of both opening up and closing down access for different groups. Hinton et al. [28] examined access to antenatal services during COVID-19; they found digital services favoured people with more ‘sociocultural capital’ who were ‘technologically adept’ over less advantaged people who lacked digital access/literacy and used ‘pay-as-you-go’ over contracted services with mobile data, as was commented upon in this study. Further, Liberati et al. [29] examined candidacy in accessing secondary mental health services during the pandemic and found people’s sense of deservedness reduced. Repeated messaging about protecting the NHS led some to perceive others as more deserving or that they would burden the NHS; fear of being perceived as not needing help prevented them seeking it. In addition, triaging has become commonplace since COVID-19; this requires people to articulate their health need convincingly before they can access treatment [29] which favours ‘more articulate, more confident and more persistent’ service users [19]. A VCSE participant in this study highlighted people’s inability and/or reluctance to advocate for their health and that of their families. This opinion corresponded with parent narratives which expressed acceptance, resignation and stoicism with regards difficulties in accessing healthcare.

Dixon-Woods et al. [19] refer to ‘appearances’ as the point at which people must assert their candidacy; once again, requiring people to give articulate and credible accounts of their needs. They also refer to ‘adjudications’ to describe the judgements of HCPs which can ‘allow or inhibit continued progression of candidacy’. Adjudications may include judgements of deservedness based on an individual’s health behaviours (e.g. diet, smoking), or about who will benefit most based on patients’ status (e.g. economically active; caring responsibilities) and likelihood of compliance with treatment [19]. Participants in this study reported difficulties in forming relationships with HCPs, lack of continuity of care was highlighted. It was also reported that people experiencing poverty often felt judged and misunderstood, consequently they may attempt to mask their financial situations. VCSE participants indicated that HCPs may misconstrue the impacts of poverty as neglect or non-compliance; they highlighted the social distance between HCPs and low-income families which contributed to such misunderstandings. Other studies report similarly, highlighting prejudice from HCPs [21], HCPs lacking appreciation of the lived realities of people experiencing poverty, resulting in inappropriate care plans, patients feeling judged for non-compliance [30] and not being able to afford the behaviours expected of them [21].

Lastly, Dixon Woods et al. [19] refer to the ‘local production of candidacy’ which will be influenced by perceived or actual availability of services locally. For example, NHS dentistry is particularly impermeable in parts of the UK, creating what have been called ‘dental deserts’. Free NHS dentistry is restricted to specific groups (e.g. under 19 years, perinatal or in receipt of welfare benefits), all others must contribute towards the costs of treatment. Notwithstanding, NHS dentistry is in crisis [31] meaning many people are unable to access regular and preventative dental care unless they can afford to pay for private care. This was highlighted by participants in our study, who lacked the financial resource to access private dental care, as a key difficulty. This is of particular concern in view of the established association between periodontal disease with systemic diseases [32].

Further, people living in the North East and North Cumbria experience differential ease of access to healthcare services depending upon where they live within the area. Some, especially those living remotely, are required to travel significant distances. Within North Cumbria there are two district general hospitals but there is no local access to tertiary care. As such, whilst NHS services may be ‘free at the point of delivery,’ when that point of delivery is many miles away there are significant issues in terms of cost (including lost income), inconvenience, travel time and challenges in terms of navigating unfamiliar buildings, places, and systems. As such, distance to healthcare creates significant barriers for low-income families, especially those living in underserved areas, in inflexible employment, with poor physical/mental health, and/or without access to a car—people who are the least able to pay.

Our analysis demonstrates how the lived experiences of low-income families attempting to navigate healthcare are inextricably linked to the cultures of healthcare systems through which stated barriers are then experienced. As Mazzoli Smith and Todd [14] found in relation to studies reflecting on the work of Poverty Proofing the School Day, an analysis of normative school cultures is inextricably bound into understanding of experiences of the school day for children living in poverty. Therefore, addressing barriers also involves addressing school culture, with positive outcomes. In this context, we have seen examples of how normative cultures of healthcare compound practical barriers, as low-income families adopt strategies and interpretive means of navigating these depending on their experiences of the cultures of healthcare systems. Such cultures include ‘poverty stigma’ [33] which compromises candidacy through the ‘poverty-shame nexus’ [34]. Consequently, experiences of navigating healthcare systems whilst living on low-incomes include both self-consciousness of one’s own position and also consciousness of the dominant views and framings of others and hence an awareness of barriers being experienced through social processes.

## Strengths and limitations

The strengths of this study lie in the voices of parent participants who shared their experiences with us, and in the narrative responses of VCSE staff who work alongside and advocate for people experiencing economic hardship. These voices add depth of understanding of the experiences—and hence causes—of known barriers to accessing healthcare and provide an important counter to understandings of barriers to accessing healthcare which do not attend to social processes and healthcare culture.

The limitations of this study are mainly methodological. We struggled to recruit parent participants which was largely due to COVID-19. Whilst COVID-19 restrictions were easing during recruitment and data collection phases, cases of COVID-19 were increasing and life was still far from ‘normal’; this may have caused anxiety for potential participants. To compensate for low parent numbers, we undertook focus groups with low-income parents attending established groups. Whilst these participants readily shared challenges experienced in accessing healthcare, we were not able to examine the impacts of these challenges on individual families in-depth nor how these challenges made them feel. The study is further limited due to the absence of HCP’s perspectives; we had hoped to talk to HCPs but this proved impossible due to health service pressures at the time.

## Conclusion

Current pressures on the UK’s NHS have resulted in access to healthcare being challenging to almost all who use it. For those living on low incomes these challenges are exacerbated in many ways, as demonstrated by this research. Our findings show that despite NHS services being free at the point of delivery, low income can be a barrier to accessing healthcare in the UK. Poverty restricts access to healthcare for children and perpetuates inequities in health, education and life opportunities. These barriers are not inevitable and through greater attention to lived experiences of barriers we are equipped with understanding about the steps that can be taken to address these.

## Supporting information

S1 Appendix COREQ checklist. (PDF)

S2 Appendix Topic guide–families. (DOCX)

S3 Appendix Topic guide–VCSE. (DOCX)

S4 Appendix Coding tree. (DOCX)
