# Supplementary material for: Poverty proofing healthcare: A qualitative study of barriers to accessing healthcare for low-income families with children in northern England
Source: PLoS One. 2024 Apr 26;19(4):e0292983. doi: 10.1371/journal.pone.0292983 (PMC11051590; doi:10.1371/journal.pone.0292983)
Supplement: S2 Appendix — (DOCX) [file pone.0292983.s002.docx]

Poverty proofing healthcare settings

**Topic guide for semi-structured interviews: families**

The purpose of this document is to provide a structure to support meaningful discussions with families on low incomes around the barriers to accessing healthcare settings. It should be used as a guide to steer conversations to help to draw out and increase understanding of the barriers faced.

**Before starting:**

- Welcome, thanks for participation and introductions
- Check that happy to record and start recording
- Verbal consent; written to follow
- Briefly remind them of the purpose of the study – part of work being done by Children North East with Newcastle University to look at ways to “poverty proof” healthcare settings like hospitals, GP surgeries etc. We want to understand what things make it difficult for people on low incomes to access healthcare and any things you think might help. Children’s North East have already done some similar work around schools, which is having a really positive impact on how children from families who are living on low incomes experience going to school. The findings from the work we’re doing now will help to make sure families are able to access the health services they need.
- Assurance that everything said will be in confidence
- Reminder that there are no ‘right’ answers – interested in opinions/experiences

| **Main theme** | Questions | Clarifying questions/probes |
| --- | --- | --- |
| **Part one**: The first part of the topic guide asks families to describe how living on a low income affects how they interact with healthcare services.  The more specific questions in part two of the topic guide can be used to “draw out” the interviewees’ experiences of interacting with healthcare services if required. | | |
| **Preamble** | We all know that lots of families are struggling to make ends meet at the moment as the cost of everything from fuel to food is increasing and that it can be challenging to find the money to make use of services like your GP and hospital, even though they are free, because there can be hidden costs associated with using them. The work I just mentioned in schools identified lots of hidden costs – like trips, lunch boxes and uniforms that Children North East have been able to help to address by changing things in schools – so things like introducing schemes for recycling school uniforms and things like that. They wouldn’t have really known about these things without speaking to a whole range of people in schools – and now we want to do the same thing with healthcare. We think that some people will find it difficult to access healthcare services because of these sort of hidden costs and we want to talk to people who use services to find out what these might be. | |
| **Understanding of “living in poverty”** | - What do you think it’s like for people living on low incomes these days? | - How do you think this might impact on people going to their doctor or going to a hospital appointment? |
| **Experience of accessing healthcare** | - Can you talk me through your own experience of accessing healthcare? | - What sort of things do you think might make it more difficult for someone living on a low income to access healthcare? |
| **Part two**: The questions below are intended to explore the views of families on specific things that have previously been identified as acting as barriers to accessing services for families living on low incomes, if these issues don’t emerge from the broader questions posed in part one. | | |
| **Appointment scheduling** | - Can you think of problems families living on a low income may encounter in attending healthcare settings due to the timing and availability of healthcare appointments? - Can you think of ways in which appointments could be better scheduled for families living on a low income? | - Is there an impact on your/your parents’ earnings due to the times when appointments are available? - Have you experienced any difficulty booking appointments because your parents’ work pattern is inflexible or you lack access to phone/internet - Is it usually possible for you to take additional people, such as siblings, to attend appointments? Does this have cost implications? |
| **Transport/travel - getting to appointments** | - What practical things might impact on the ability of families living on a low income to get to appointments or seek healthcare? - Can you think of ways in which families could be supported to access healthcare? | - How do you think the costs of petrol/parking/public transport might prevent families attending? - To what extent do you think that distance from home might be an issue for families? - To what extent do you think accessibility by public transport an issue? |
| **Getting a diagnosis** | - Have you been in a situation where you have needed to attend multiple appointments to get a diagnosis or because you have a condition that requires ongoing care? - If so, are there specific difficulties you face due to this? - In what ways do you think that families living on a low income who have children who need multiple appointments to reach a diagnosis or have a condition that requires ongoing care face problems getting healthcare? |  |
|  |  |  |
| **Admission/discharge** | - Can you describe what affordable food is available (or food provided free of charge) for families attending healthcare appointments? - Have you any experience of being admitted to hospital out of hours? Did this present any particular problems for you? - Was 'equipment', such as nightwear and toiletries, available for overnight stays for you? - Have you any experience of being discharged from hospital out of hours (e.g. late at night?). Did this present any particular problems for you? - Can you describe ways in which these things may create problems for families living on a low income? |  |
| **Communication** | - In what way is information about your health and the treatments they need communicated to families? - Can you think of ways in which information could be shared better with families? | - To what extent did you find the language used easy to understand? |
|  |  |  |
|  | - Is there any information about your circumstances that you think it would be helpful for healthcare professionals to share with other professionals you are likely to come into contact with? - Can you describe ways in which these things may create problems for families living on a low income? | - Would it, for example, be helpful if staff within a new service you access knew about your family circumstances? |
| **Relationships** | - To what extent do you feel that you build trusting relationships with staff? - Can you think of ways in which staff might develop better relationships with families? - Can you describe reasons why families living on a low income might struggle to form trusting relationships with staff? - Can you describe ways in which staff might develop better relationships with families living on a low income? | - Do you know of any particular groups of healthcare staff who are approachable? - What do these staff do to make sure they are approachable? |
| **What have we missed?** | Are there any other problems families living on a low income might experiencing in getting health care? |  |
|  |  |  |
