# Supplementary material for: Poverty proofing healthcare: A qualitative study of barriers to accessing healthcare for low-income families with children in northern England
Source: PLoS One. 2024 Apr 26;19(4):e0292983. doi: 10.1371/journal.pone.0292983 (PMC11051590; doi:10.1371/journal.pone.0292983)
Supplement: S3 Appendix — (DOCX) [file pone.0292983.s003.docx]

Poverty proofing healthcare settings

**Topic guide for semi-structured interviews/focus groups: Community Groups**

The purpose of this document is to provide a structure to support meaningful discussions with professionals/practitioners from non-healthcare settings (e.g. community groups) around the barriers to accessing healthcare settings faced by people living in poverty. It should be used as a guide to steer conversations to help to draw out and increase understanding of the barriers faced.

| **Main theme** | **Questions** | **Clarifying questions/probes** |
| --- | --- | --- |
| **Part one**: The first part of the topic guide asks professionals/practitioners to talk in general about their understanding of what living in poverty means and how living on a low income might affect how a family interacts with healthcare services.  The more specific questions in part two of the topic guide can be used to “draw out” the interviewees’ experiences of supporting families living in poverty if required. | | |
| **Families living in poverty** | - What does 'living in poverty' mean to you? - Would you typically be aware that a family accessing your service was living in poverty or are there occasions when you would not know? - How do you think families living in poverty are judged by other people? | - Can you describe how you would become aware that a family accessing your service was living in poverty? |
| **Understanding of how people living in poverty experience accessing healthcare** | - What sort of barriers do you think there are that might make it more difficult for someone living in poverty to access healthcare? - It might be helpful to think of a ‘case study’ here – i.e. draw on your experience of working with an individual family and what their journey through the healthcare system might look like. | - Can you describe how a family living in poverty might experience accessing healthcare services? - E.g. A GP appointment, a hospital appointment, an in-patient stay |
| **Part two**: The questions below are intended to explore the views of professionals/practitioners on specific things that have previously been identified as acting as barriers to accessing services for families living on low incomes, if these issues don’t emerge from the broader questions posed in part one. | | |
| **Appointment scheduling** | - Do you think families living in poverty might encounter difficulties in attending healthcare settings due to the timing and availability of healthcare appointments? - Can you think of ways in which appointments could be better scheduled for families living in poverty? | - What might be the impact on parents/carers earnings due to the times when appointments are available? - What issues do you think there may be in relation to appointment times for some families? - To what extent do you agree that it is more difficult for some groups to book convenient appointment times (e.g. because of lack of access to phone/internet) than others? - To what extent do you think provision is made for additional individuals, such as siblings, to attend appointments – any cost implications of this? |
| **Transport/travel - getting to appointments** | - What practical things might impact on the ability of families living in poverty to attend appointments or seek healthcare? | - How do you think the costs of petrol/parking/public transport might prevent families attending? - To what extent do you think that distance from home might be an issue for families? - To what extent do you think accessibility by public transport an issue? |
|  | - Do you know of any discounts or sources of support with travel costs that families could access? - To what extent do you think families know about discounts, support or exemptions for travel? - In what ways do you think families living in poverty could be supported to access healthcare? | - Which groups do you think are less able to benefit from discounts/exemptions (e.g. because of lack of access to phone/internet) |
| **Diagnostic process** | - In what ways do you think that families living in poverty with children who need multiple appointments to reach a diagnosis or have a condition that requires ongoing care face specific problems? | - To what extent are families made aware of the likely sequence of appointments? - In what ways is the information communicated? |
|  |  |  |
| **Admission/discharge** | - Can you describe what affordable food is available (or food provided free of charge) for families attending healthcare appointments or spending long periods of time at the hospital due to the type of treatment required? - Can you describe what happens when a patient is admitted out of hours? What problems might this cause for families living in poverty? - Is 'equipment', such as nightwear and toiletries, available for overnight stays for children and for parents/carers? - Can you describe what happens when patients are discharged out of hours (e.g. late at night?). Might this cause problems for families living in poverty? |  |
| **Communication** | - Is there any particular information that you think should be shared between professionals about families living in poverty? - In what way is information about health conditions, diagnoses and treatments communicated to families? - Can you think of ways in which information could be shared better with families? | - To what extent do you think families understand the language and terminology used? - In what ways do you think staff consider a family's circumstances when communicating with them? |
|  |  |  |
|  | - What kind of information is shared about families between healthcare staff? | - Are details of the family's circumstances (e.g. living conditions, family size, social needs) shared? - Are there ways in which information about economic circumstances of families could be shared more effectively? - How would sharing of this information influence practice? |
| **Relationships** | - To what extent do families build trusting relationships with staff? - Can you describe reasons why families living in poverty might struggle to form trusting relationships with staff? - Can you describe ways in which staff might develop better relationships with families living in poverty? | - Do you know of any particular groups of healthcare staff who are approachable/make time to nurture these relationships? - What do these staff do to make sure they are approachable? |
| **Staff awareness/attitudes** | - To what extent do staff understand issues faced by families living in poverty? | - Are there indicators of financial difficulty that staff look out for? |
|  | - Have you experience of staff expressing opinions (negative or positive) about families living in poverty? | - Where would staff signpost families for support if needed? - What strategies, if any, are used to make sure support is provided tactfully? - How could staff be supported to better understand issues faced by families living in poverty? |
