# Supplementary material for: Poverty proofing healthcare: A qualitative study of barriers to accessing healthcare for low-income families with children in northern England
Source: PLoS One. 2024 Apr 26;19(4):e0292983. doi: 10.1371/journal.pone.0292983 (PMC11051590; doi:10.1371/journal.pone.0292983)
Supplement: S4 Appendix — (DOCX) [file pone.0292983.s004.docx]

**Barriers to Healthcare access – coding tree**

**Hidden costs**

Transport

Public transport

Taxis

Parking

Distance to services

Subsistence

Loss of income/employment

**Securing Appointments**

Lack of appointments

GP

Dentist

Exacerbating conditions

Appointment booking systems

Digital

Telephone

Timing of appointments/opening hours

**Navigating the system**

Advocating for self and family

Stress

No childcare

Acceptance/giving up/not trying

A&E

**Trusting relationships with HCPs/providers**

Lack of continuity in care

HCPs misunderstanding family circumstances

Social distance

Poor communications

Good experiences

**Specific health services**

Dentists

Lack of dentists/appointments

Children in pain

GP

Appointment times

Getting an appointment

Telephoning

Health visitors

Hospitals

Appointment times

Emergency admissions

Discharge

Subsistence

Mental health

Securing referrals

Service pressures

**Rurality/remote**

Distance to services

Increased costs

Delayed care

Prefer to travel to tertiary centre

**Life on a low income**

Emotional effects

Stress

Embarrassment

Stigma

Feeling judged

Health effects

Diet

Mental health

Social effects

Limits choices

Social isolation

Misunderstood

**Health provider responses**

Social distance

Lack of awareness

**Financial assistance**

Accessed but

No awareness

Poor information

**Improvements**

Increase knowledge on financial assistance

Help with subsistence costs

System changes

Bookable appointments

More choice over appointment times

Training for HCPs on impacts of poverty

Placements in deprived areas

Sharing information on family circumstances
